# Oil–water partition coefficient preparation and detection in the dihydroartemisinin self-emulsifying drug delivery system

**DOI:** 10.1186/s12896-022-00746-6

**Published:** 2022-05-27

**Authors:** Yunhong Wang, Jingcai Chen, Yang Yang, Sijia Gao, Zhuzhu Wang, Yating Liu, Xiaomei Zhang, Lei Hua, Yanlei Guo, Yong Yang

**Affiliations:** 1 Chongqing Academy of Chinese Materia Medica, No.34 of Nanshan Road, Nanan District, Chongqing, 400065 China; 2 Chengdu University of TCM, Chegndu, 611137 China; 3 Chongqing University of Education, Chongqing, 400065 China

**Keywords:** Dihydroartemisinin, SEDDS, Ternary phase diagram, Central composite design-response surface methodology, Oil–water partition coefficient

## Abstract

**Background:**

The aim of the present study is to increase the solubility of dihydroartemisinin (DHA) using the self-emulsifying drug delivery system (SEDDS).

**Methods:**

We first conducted solubility test and ternary phase diagram, then, in order to optimize the formulation of the DHA self-emulsifying agent, the design mixture method was selected in the design expert software. Next, optimal prescription validation and preliminary formulation evaluation were conducted. By comparing the oil–water partition coefficient in vitro, the improvement of the in vivo osmotic absorption of DHA via self-emulsification was evaluated.

**Results:**

The optimal prescription ratio of oleic acid polyethylene glycol glyceride, polyoxyethylene hydrogenated castor oil, and diethylene glycol monoethyl ether in the DHA self-emulsifying preparation = 0.511:0.2:0.289 (w/w/w), with a drug-loading capacity of 26.3634 mg/g, solubility of 2.5448 mg/ml, and self-emulsification time of 230 s. The solubility self-emulsification was approximately 20.52 × higher in DHA than in the crude drug. The self-emulsification could improve DHA permeability and promoting in vivo DHA absorption.

**Conclusion:**

The DHA SEDDS could significantly improve DHA solubility and in vivo absorption.

## Background

A significant percentage (up to 70%) of chemical substances considered in drug development has poor aqueous solubility problem that will affect gastrointestinal absorption. A renowned alternative approach for delivery of the low water-soluble drug is by formulating as a lipid formulation particularly the self emulsifying drug delivery systems (SEDDS) which deal with low aqueous solubility and poor oral bioavailability [1]. A renowned alternative approach for delivery of the low water-soluble drug is by formulating as a lipid formulation particularly the selfemulsifying drug delivery systems (SEDDS) which deal with low aqueous solubility and poor oral bioavailability [1].

Surfactant, oil-phase, and drug-isotropic mixtures are self-emulsifying drug delivery systems (SEDDS). When they encounter a water-soluble medium, oil-in-water emulsion droplets can be rapidly formed by mild agitation or digestive movement under gastrointestinal conditions [2]. Self-emulsifying drug drops can be divided into two types according to their size: (1) the self-microemulsifying drug delivery system and (2) the self-nanoemulsifying drug delivery system.

There are many advantages to the use of SEDDS, including physical stability, a simple manufacturing process, and oral application via soft or hard gelatin capsules underline the intensive research conducted within the last decades [3–6].

DHA is soluble in acetone, slightly soluble in methanol or ethanol, and almost insoluble in water. The solubility in water was determined to be 0.124 mg/ mL after shaking for 24 h in a 37 ℃ constant temperature oscillating chamber. Dihydroartemisinin (DHA) is obtained from the reduction of artemisinin using sodium tetrahydroborate. Its structure is characterized by a unique peroxide bridge; this has various advantages (e.g., high potency, micro-toxicity, rapid excretion, metabolism, and absorption by the human body, and wide distribution). In addition, the antimalarial effect of DHA is 4 − 8 × greater than that of artemisinin [7]. However, the solubility of DHA in water is relatively low; thus, the present study aims to improve DHA solubility through self-emulsifying formulation.

## Material and methods

### Reagents, and drugs

The DHA reference substance (National Institutes for Food and Drug Control, Batch number: 100184–201403); oleic acid polyethylene glycol glyceride (Oleoyl Macrogolglycerides, batch number: M01GS147525, Yuanye Bio-Technology Co. Ltd); polyoxyethylene hydrogenated castor oil (Cremophor RH40, Batch number: Y23M10S83793, Shanghai Yuanye Bio-Technology Co. Ltd); Transcutol P (Batch number: 177546, Tianrun Pharmaceutical Co.); and DHA bulk pharmaceutical chemicals (Chongqing Wulingshan KPC Pharmaceuticals Inc, batch number: C00220181001, content 95%). The methanol was chromatographically pure, and the other items were analytically pure.

## Methods

### Determination method for DHA content

Chromatographic conditions: Target C18(2) (250 × 4.6 mm, 5 mm), mobile phase: acetonitrile–phosphate buffer solution (1.36 G of monopotassium phosphate was injected, dissolved in 900 ml of water, and pH was adjusted to 3.0 using phosphoric acid. The final volume was obtained and injected with 1000 mL of water) (44:56). Detection: (1) Wavelength: 216 nm; (2) flow rate: 1 mL/min; (3) column temperature: 35 °C; and (4) injection volume: 10 μL.

Reference solution preparation: An appropriate amount of DHA reference control was weighed and dissolved in acetonitrile, and the solution was shaken well.

Test solution preparation: A volume of 0.08 ± 0.01 g of the DHA self-emulsifying preparation was weighed precisely and placed in a 5 mL measuring bottle. Next, the preparation was dissolved in acetonitrile and diluted to the scale. The solution was shaken for further detection using SK-1 quick mixer (Jintan East City Xinrui Instrument Factory).

Blank solution preparation: A volume of 0.08 ± 0.01 g of blank self-emulsifying preparation without DHA was weighed precisely and placed in a 5 mL measuring bottle. Next, the preparation was dissolved in acetonitrile, diluted to the scale, and shaken well.

**Fig. 1:**
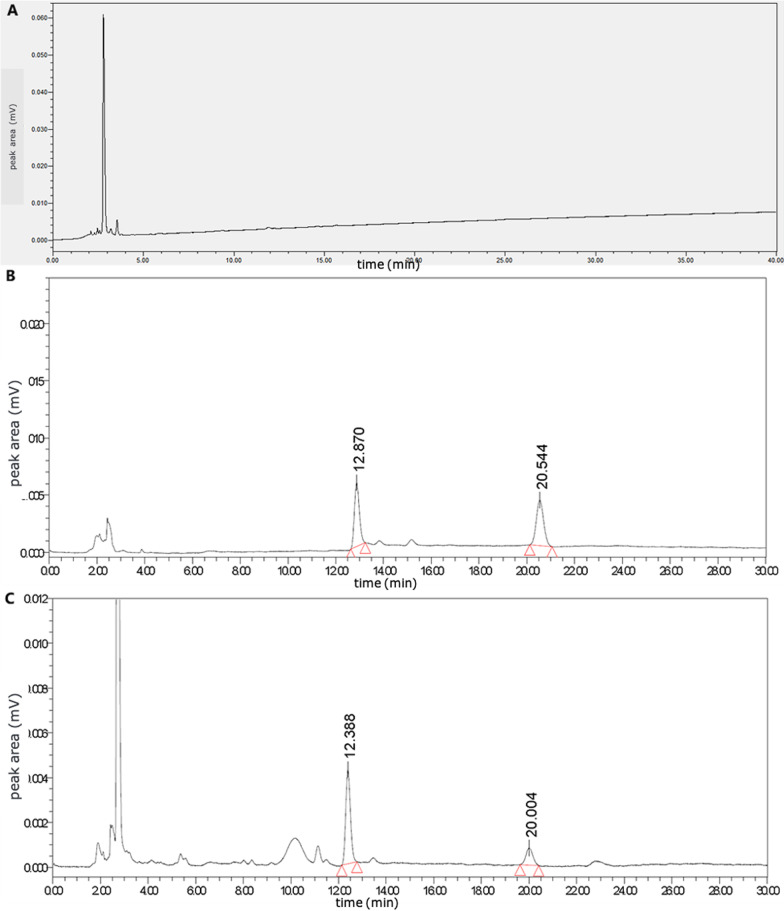
The HPLC chromatograms of the blank solution **A**, dihydroartemisinin reference solution **B**, and test solution **C**

Method specificity inspection: The blank solution, DHA control solution, and test solution, respectively, were taken and determined according to the above chromatographic conditions (Fig. 1). As demonstrated in Fig. 1, the ingredients did not interfere in the detection of DHA.

Determination of linear correlation: The DHA control solution was used to prepare a series of control solutions for the peak area measurement and recording. Linear regression x coordinates (x) were used as the mass concentration (μg/ml), and y coordinates (y) were used as the peak area. The standard curve equation was y = 255.65 x − 1453.9 (*r* = 0.99999). The results showed that the DHA concentration had a good linear correlation with the peak area within a range of 150.46 ~ 3009.2 μg/mL.

Precision test: The stability, repeatability, and sample recovery of the reference samples were simultaneously tested, and the solution and test solution, respectively, were conducted within 24 h. All parameters met the study requirements, indicating that the method was accurate. Both the reference solution and the test solution became stable within 24 h.

### Preparation of the self-emulsifying DHA formulation

#### Screening of the blank self-emulsification prescription

##### Determination of DHA solubility in various ingredients

Approximately 2 mL of different oil phases, surfactants, and co-surfactants, respectively, was taken and placed into plug test tubes. Excessive amounts of the crude DHA drug were added and vortexed; the tubes were shaken at 37 °C on a constant temperature oscillation (HZ-881S desktop water bath thermostatic oscillator (Jiangsu Taicang Experimental Equipment Factory)) box for 24 h and centrifuged at 10,000r/min for 10 min (Dongfeng-101 s constant temperature heating collector magnetic stirring instrument (Zhengzhou Greatwall Scientific Industrial and Trade Co, Ltd), RC806 dissolution experimental instrument (Tianjin Tianda Tianfa Technology Co., Ltd)). The required test solution was prepared in accordance with the prescribed procedure.

##### Ternary phase diagram construction

In the present study, the screening range of each phase in the self-emulsification prescription was limited as follows: (1) the oil phase: 20%–80%; (2) the surfactant phase: 20–80%; and (3) the co-surfactant phase: 0%–30%. Based on the above composition ranges, different proportions of oil phases, surfactants, and co-surfactants were weighed and mixed using a vortex.

The mixtures were then kept at room temperature for 24 h; the occurrence of stratification was observed, and the proportion with stratification was discarded. The emulsifying process was observed and recorded after injecting 100 ML of water into a 0.5 ml non-layered prescription at (37 ± 2) °C during magnetic stirring. With the surfactants, co-surfactants, and oil phases as one side, the proportion that could form clear and transparent oil droplets without floating was determined as the effective self-emulsification region in the phase diagram, and the ternary phase diagram was constructed.

#### Prescription optimization using the mixture-optimal (custom) design

Based on the ternary phase diagram, the Mixture-Optimal (Custom) design created using Design Expert 11 was adopted for composition optimization. According to the scope of the investigation, the oleic acid polyethylene glycol glyceride (CS, A) range was set at 20–80%, the polyoxyethylene hydrogenated castor oil (EL, B) range was set at 20–80%, and the diethylene glycol monoethyl ether (YP, C) range was set at 0–30%. DHA SEDDS particle size was measured by laser particle size analyzer, DHA SEDDS about 0.2 g, add water to 5 mL, shake well, determination.

### Determination of the apparent oil–water partition coefficient

The shaking flask method was used for determination [8, 9]. A proper amount of DHA was dissolved in water-saturated N-octanol, the 1.2 pH hydrochloric acid solution, and the 4.5, 6.8, and 7.4 pH phosphate buffers, respectively.

Thus, a series of drug-saturated N-octanol solutions were prepared. A volume of 1.0 mL of each of the above solutions was measured precisely and put into 5 plug test tubes; next, 4 mL of water and the corresponding N-octanol-saturated pH buffer were successively added. After vortexing for 5 min and shaking at (37 ± 2) °C on a constant temperature oscillation box for 24 h, the tubes were taken out and kept standing still for 30 min. After this, the two phases were separated by 10,000 r/min centrifugation for 10 min. The water intake layer and the alcohol layer were checked. The concentration of DHA and the logP_app_ were calculated. The calculation formula was as follows:

$$p_{app} = \frac{Coil}{{C_{water} }} = \frac{{4 \times \left( {C_{t} } \right)}}{{C_{0} - C_{t} }}$$

In the above equation, P_app_ was the apparent oil–water partition coefficient; C_0_ was the initial concentration of the drug in N-octanol; and Ct was the concentration measured in the oil phase at the equilibrium partition of the drug.

## Results

**Fig. 2:**
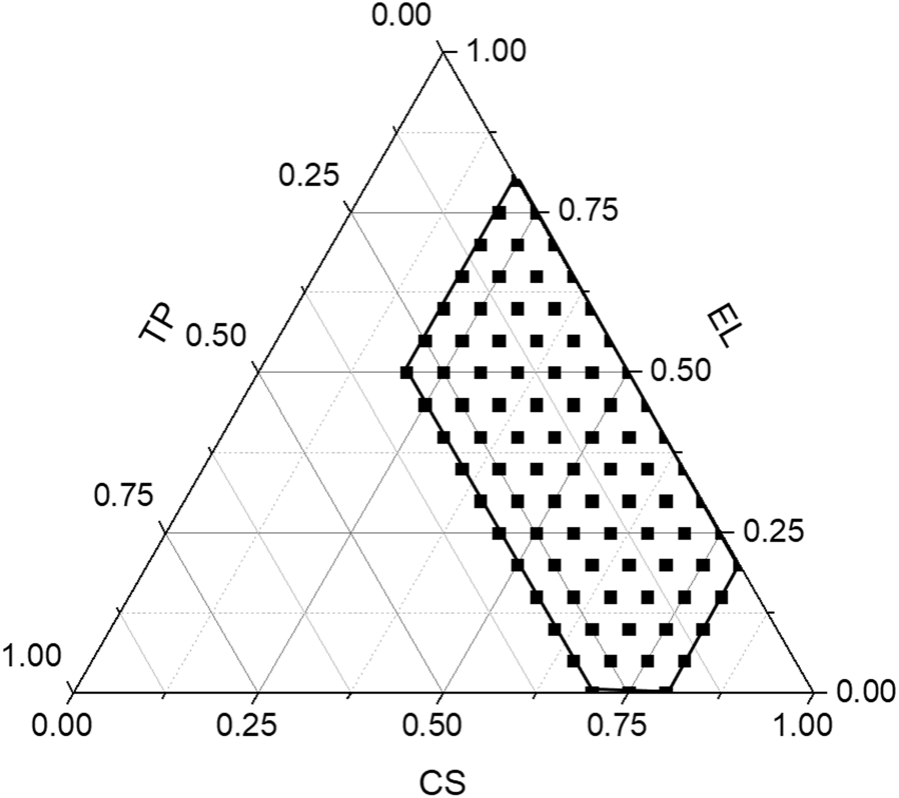
The ternary phase diagram

**Fig. 3:**
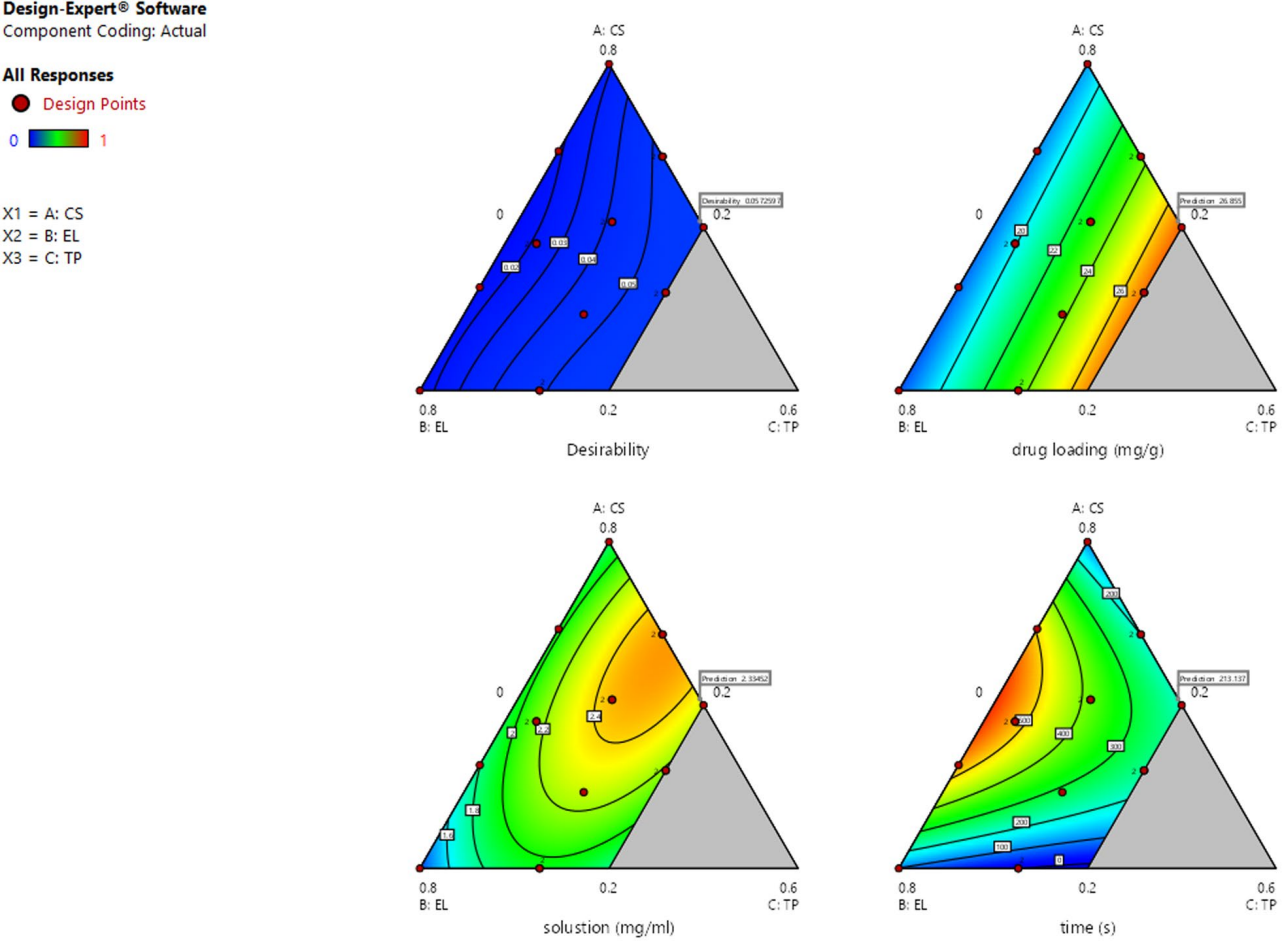
The contour map

**Fig. 4:**
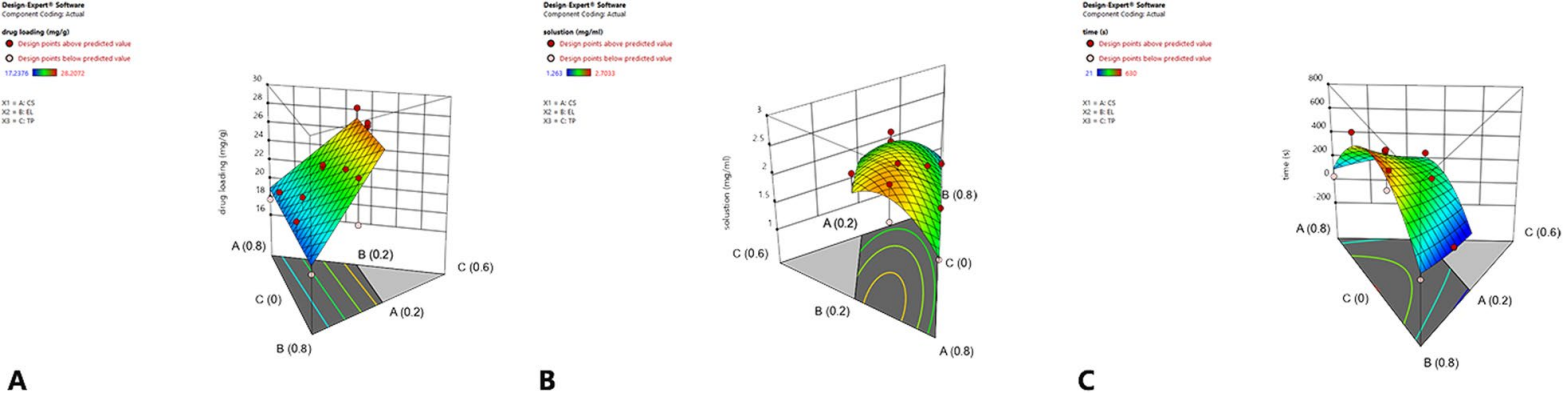
The influence of prescription composition on each indicator. **A** Three-dimensional effect diagram of the influence of prescription composition on drug loading. **B** Three-dimensional effect diagram of the influence of prescription composition on solubility. **C** Three-dimensional effect diagram of the influence of prescription composition on emulsification time

1. Determination of DHA solubility in various ingredients
  The solubility of DHA in different components was determined and shown in Table 1.
2. Ternary phase diagram construction
  The composition of self-emulsification was as follows: oleic acid polyethylene glycol glyceride (CS)—polyoxyethylene hydrogenated castor oil (EL)—diethylene glycol monoethyl ether (TP). The ternary phase diagram is shown in Fig. 2; the black dots represent the test points, and the area inside the black line represents the effective self-emulsification area. The results showed that emulsification could be achieved within the range of investigation.
3. Prescription optimization using the Mixture-Optimal (Custom) design
  The DHA drug load, self-emulsification time, and emulsification time were taken as indicators in the design. The design, experimental factors, and results are shown in Tables 2 and 3. Oleic acid polyethylene glycol glyceride, polyoxyethylene hydrogenated castor oil, and diethylene glycol monoethyl ether were weighed according to the central composite design table. Excessive crude DHA drug was added for mixing and vortexing, and the drug loading was determined. A volume of 200 μL of each test site’s samples was taken and placed into 2 ml of water. Excessive crude DHA drug was added and shaken at 37 °C on a constant temperature oscillation box for 24 h. The mixture was then filtered, and the solubility was measured. At the same time, the 0.2 ml emulsion containing the drug was added to 200 ML of water at a temperature of 37 °C, and the dissolution was determined using the dissolution apparatus. Self-emulsification was achieved by stirring slightly at a rotating speed of 50 r/min with the paddle method, and the emulsification time was recorded with a stopwatch using the visual method.
  Model fitting: The Design Expert 11 software was adopted for data model fitting. The fitting model regression equations were as follows: (1) drug loading = 19.22093 A + 17.80913 B + 46.64294 C; (2) solubility = 1.39553 A + 0.61703 B − 7.86810 C + 3.67178 A*B + 17.34126.
  A*C + 14.44074 B*C; and (3) time =  − 798.47284 A − 801.88714 B + 79.06520 C + 5613.82544 A*B + 1626.57588 A*C − 956.46250 B*C. The fittings of each indicator are illustrated in Table 4.
4. The contour map (Fig. 3) and effect surface 3D diagram (Fig. 4) concerning the three evaluation indicators and the influences of the three kinds of ingredients were obtained from the Mixture-Optimal (Custom) design.
5. Prediction, validation, and preliminary evaluation of the optimal prescription
  (1) Prediction and validation of the optimal prescription: In the present study, the prescription composition of the self-emulsification DHA preparation was optimized with a large drug load, maximum solubility, and shortest self-emulsification time.
  The predicted optimal prescription ratio was as follows: oleic acid polyethylene glycol glyceride to polyoxyethylene hydrogenated castor oil to diethylene glycol monoethyl ether = 0.511:0.2:0.289 (w/w/w). Predicted drug load = 26.8507 mg/g; solubility = 2.33503 mg/ml; and self-emulsification time = 213.148 s.
  The self-emulsifying prescription was prepared with the predicted optimal formula ratio, and the drug load, solubility, and self-emulsification time were determined. The absolute deviation of each indicator was < 10%; this confirmed the good prediction of the mathematical model. The results are demonstrated in Table 5.
6. Other quality evaluations: (1) Appearance: The DHA blank control and the SEDDS containing the drug appeared transparent, with the ingredients slightly yellow in color, and were in the form of oil; (2) physical stability: the DHA SEDDS was centrifuged at 4,000 r·min^−1^ for 15 min, and no stratification was observed, indicating good physical stability; and (3) particle diameter: the DHA SEEDS was diluted 25 × with water. According to the experimental results, the average particle size of the emulsifier = 136.3 nm. The average Zeta potential of the optimal prescription was -4.13 mV.
7. Results of the oil–water partition coefficient

**Table 1:** The solubility of dihydroartemisinin in different oil phases, emulsifiers, and co-emulsifiers

Oil phase	Solubility	Surfactant	Solubility	Co-surfactant	Solubility
Medium chain triglyceride	2.578	Tween-20	15.702	Glycerin	31.520
Glyceryl monooleate	8.478	Tween-40	9.379	Polyethylene glycol	8.170
Soybean oil	5.130	Tween-60	6.571	Diethylene glycol ether	17.083
Corn oil	1.829	Tween-80	8.665	Diethylene glycol monoethyl ether	46.679
Olive oil	2.259	Span-80	10.966	1,2-Propanediol	3.637
Castor oil	2.468	Isopropyl myristate	2.093	Isopropanol	9.364
Ethyl oleate	5.525	Triethanolamine	4.740		
Oleic acid polyethylene glycol glyceride	12.113	Span-85	1.182		
Oleic acid	1.954	Polyethylene glycol monooleate	11.326		
		Castor oil polyoxyethylene ether	12.573		
		Isopropyl palmitate	0.890		
		Oleoyl polyoxyethylene glyceride	8.256		
		Polyethylene glycol-7-stearate	3.324		
		Polyoxyethylene hydrogenated castor oil	6.518		
		Caprylic acid capric acid polyethylene glycol glyceride	32.558

**Table 2:** The factors and levels of mixture-optimal (custom)

	Name	Low	High
A	CS	0.2	0.8
B	EL	0.2	0.8
C	TP	0	0.3

**Table 3:** The design and results of Mixture-Optimal (Custom)

	A:CS	B:EL	C:TP	Drug loading (mg/g)	Solustion (mg/ml)	Time (s)
1	0.64	0.36	0	20.2264	2.0550	609
2	0.63	0.2	0.17	21.9424	2.0729	266
3	0.2	0.8	0	17.2376	1.2630	46
4	0.5	0.2	0.3	28.2072	2.6206	250
5	0.38	0.32	0.3	27.3796	2.0002	200
6	0.34	0.47	0.19	24.2283	2.2242	388
7	0.2	0.61	0.19	24.8118	1.9490	21
8	0.2	0.61	0.19	20.2574	2.1184	25
9	0.63	0.2	0.17	22.6987	2.7033	247
10	0.38	0.32	0.3	27.6726	2.0003	194
11	0.51	0.34	0.15	23.2940	2.4315	274
12	0.39	0.61	0	20.0242	1.9104	630
13	0.47	0.48	0.05	19.0424	2.2363	298
14	0.8	0.2	0	17.7117	1.8063	35
15	0.47	0.48	0.05	21.1384	1.8877	621
16	0.51	0.34	0.15	23.0316	2.5780	313

**Table 4:** The fitting table of various indicators

Response	Model	Source	Sum of squares	df	Mean square	F-value	*p*-value	
Drug loading	Linear model	*Model*	145.66	2	72.83	34.11	< 0.0001	Significant
		Linear mixture	145.66	2	72.83	34.11	< 0.0001	
		*Residual*	27.76	13	2.14			
		Lack of fit	14.83	8	1.85	0.72	0.6789	Not significant
		Pure error	12.93	5	2.59			
		*Cor total*	173.42	15				
Solustion	Linear model	*Model*	1.38	5	0.28	5.10	0.0139	Significant
		Linear Mixture	0.84	2	0.42	7.81	0.0091	
		AB	0.16	1	0.16	2.94	0.1170	
		AC	0.37	1	0.37	6.93	0.0250	
		BC	0.33	1	0.33	6.12	0.0329	
		*Residual*	0.54	10	0.054			
		Lack of fit	0.26	5	0.051	0.90	0.5460	Not significant
		Pure error	0.28	5	0.057			
		*Cor total*	1.92	15				
Time	Quadratic model	*Model*	4.931E + 05	5	98,626.17	7.62	0.0034	Significant
		Linear Mixture	93,897.03	2	46,948.52	3.63	0.0654	
		AB	3.714E + 05	1	3.714E + 05	28.69	0.0003	
		AC	3293.05	1	3293.05	0.2544	0.6249	
		BC	1448.40	1	1448.40	0.1119	0.7449	
		*Residual*	1.294E + 05	10	12,943.19			
		Lack of fit	76,315.64	5	15,263.13	1.44	0.3503	Not significant
		Pure error	53,116.25	5	10,623.25			
		*Cor total*	6.226E + 05	15

**Table 5:** Validation of the optimal prescription (*n* = 3)

Indicator	The predictive value	The actual value	The deviation
The drug loading content (mg/g)	26.855	26.3634	1.83
The solubility (mg/mL)	2.335	2.5448	− 8.99
The emulsification time (s)	213.148	230	− 7.9

Deviation% = (The predictive value − The actual value)/ The predictive value × 100%

Theoretically, the logP_app_ value reflects the lipophilicity and hydrophilia of the drug: the larger the logP_app_ value, the higher the lipophilicity, the lower the logP_app_ value, the higher the hydrophilia, and the smaller the hydrophilic. When logP_app_ < 0, drug absorption in the gastrointestinal tract is very difficult. When 0 < logPapp < 3, drugs can be absorbed by the gastrointestinal tract. When logP_app_ > 3, drugs have strong lipid solubility and are not conducive to gastrointestinal absorption [10, 11].

As illustrated in Table 6, the logP_app_ of the crude DHA drug = 0–3 when water was adopted as the medium. The logP_app_ values of the crude drug were all < 0 when other pH buffers were adopted as the medium. In the solutions with different pH adopted as the medium, the logP_app_ of the DHA self-emulsifying preparation = 0–3, indicating that the DHA self-emulsifying preparations were more absorbable by the body than the crude drugs.

**Table 6:** The results of the apparent oil–water partition coefficient of dihydroartemisinin crude drug

The solvent	The crude drug		SEDDS	
	P_app_	logP_app_	P_app_	logP_app_
Distilled water	− 64.8182	− 1.8117	29.0999	1.4639
Hydrochloric acid solution pH 1.2	− 12.4463	− 1.0950	22.6545	1.3552
Phosphate buffer pH 4.5	− 1.3144	− 0.1187	43.9862	1.6433
Phosphate buffer pH 6.8	− 1.1964	− 0.0779	24.8717	1.3957
Phosphate buffer pH 7.4	− 47.8224	− 1.6796	20.6708	1.3154

## Discussion

In the preparation of self-emulsifying DHA formulations, the self-emulsifying combinations were conducted according to the solubility of DHA in each phase. The following combinations were investigated: glyceryl mono-oil-Tween 20-glycerol; ethyl oleate, castor oil polyoxyethylene ether, and diethylene glycol ethyl ether; soybean oil, polyethylene glycol monooleate, and isopropanol; oleic acid ethyl ester-Span 85-isopropanol; castor oil-Tween 80-diethylene glycol ethyl ether; ethyl oleate, glyceryl triacetate, and isopropanol; ethyl oleate, isopropyl myristate, and diethylene glycol ethyl ether; medium-chain triglyceride-castor oil polyoxyethylene ether-isopropanol; soybean oil, Tween 40, and glycerin; medium-chain triglyceride-glyceryl triacetate-diethylene glycol ethyl ether; glycerol monooleate-Tween 20-diethylene glycol ethyl ether; ethyl oleate-castor oil polyoxyethylene ether-glycerin; ethyl oleate-Tween 80-diethylene glycol ethyl ether; medium-chain triglycerides-Tween 80-polyethylene glycol 400; medium-chain triglyceride-Tween 40-diethylene glycol ethyl ether; isopropyl myristate-Tween 40-diethylene glycol ethyl ether; ethyl oleate-Tween 40-diethylene glycol ethyl ether; and polyethylene glycol glyceryl oleate-polyoxyethylene hydrogenated castor oil-diethylene glycol monoethyl ether.

However, as the ternary phase diagram in the three phases failed to achieve the formation of emulsification, or the area of the emulsion was too small, ingredients with non-maximum solubility were selected in the investigation. In vivo pharmacokinetic studies, it was found that DHA would be metabolized after entering the body, and artemisinin and artesunate components could be detected simultaneously. The metabolism of DHA in vivo needs further study.

DHA has two peaks in high-performance liquid chromatography; the sum of the two peak areas was adopted to calculate the content [12]. In the follow-up study, investigation of the two peaks’ transformation patterns should be continued in order to provide a basis for further study.

## Conclusions

The optimized dosage of DHA SEDDS was 26.3634 mg/g, the solubility was 2.5448 mg/ mL, the self-emulsification time was 230 s, the average particle size was 136.3 nm, and the average Zeta potential was -4.13 mV. Self-emulsification increased the solubility of dihydroartemisinin by about 20.52 times compared with the bulk drug, and the apparent oil–water partition coefficient predicted that SEDDS could improve the absorption of DHA in vivo.

## Data Availability

The datasets generated and/or analyzed during the current study are not publicly available due to the authors’ decision but are available from the corresponding author on reasonable request.

## Abbreviations

SEDDS: Self-emulsifying drug delivery systems
DHA: Dihydroartemisinin
